# A stochastic model to investigate the effects of control strategies on calves exposed to *Ostertagia ostertagi*

**DOI:** 10.1017/S0031182016001438

**Published:** 2016-08-30

**Authors:** ZOE BERK, YAN C. S. M. LAURENSON, ANDREW B. FORBES, ILIAS KYRIAZAKIS

**Affiliations:** 1 School of Agriculture Food and Rural Development, Newcastle University, Newcastle upon Tyne NE1 7RU, UK; 2 Animal Science, School of Environmental and Rural Science, University of New England, Armidale, New South Wales 2351, Australia; 3 Scottish Centre for Production Animal Health and Food Safety, School of Veterinary Medicine, University of Glasgow, Glasgow G61 1QH, Scotland

**Keywords:** calves, nematodes, management, *Ostertagia ostertagi*, parasite control, phenotypic variation, simulation, mathematical model

## Abstract

Predicting the effectiveness of parasite control strategies requires accounting for the responses of individual hosts and the epidemiology of parasite supra- and infra-populations. The first objective was to develop a stochastic model that predicted the parasitological interactions within a group of first season grazing calves challenged by *Ostertagia ostertagi*, by considering phenotypic variation amongst the calves and variation in parasite infra-population. Model behaviour was assessed using variations in parasite supra-population and calf stocking rate. The model showed the initial pasture infection level to have little impact on parasitological output traits, such as worm burdens and FEC, or overall performance of calves, whereas increasing stocking rate had a disproportionately large effect on both parasitological and performance traits. Model predictions were compared with published data taken from experiments on common control strategies, such as reducing stocking rates, the ‘dose and move’ strategy and strategic treatment with anthelmintic at specific times. Model predictions showed in most cases reasonable agreement with observations, supporting model robustness. The stochastic model developed is flexible, with the potential to predict the consequences of other nematode control strategies, such as targeted selective treatments on groups of grazing calves.

## INTRODUCTION

Gastrointestinal parasitism of calves, in particular with *Ostertagia ostertagi*, is a significant challenge to their health, welfare and productivity. As such, a variety of control strategies have been proposed to reduce the negative effects of parasitism (Cockroft, 2015). These include the Weybridge ‘dose and move’ strategy, a reduction in stocking rate and dosing at strategic time points of the grazing season (Michel and Lancaster, 1970; Hansen *et al.* 1989; Cockroft, 2015). More recently, targeted selective treatment, where specific individuals of a population as opposed to the whole population are treated, has been suggested as an alternative control strategy (Höglund *et al.* 2013*a*; O'Shaughnessy *et al.* 2015).

Quantifying the effectiveness of such strategies is both time consuming and expensive, and in many respects it is difficult, if not impossible to make comparisons between them due to confounding variables (Höglund, 2010). Simulation modelling is a potential alternative to experimentation and, provided that a model is based on sound principles and data, it has the potential to evaluate different approaches to control. In order to be able to assess the effectiveness of such control strategies, a stochastic (i.e. probabilistic, population-based) model allowing for individual-response differences is required. This is because individuals will affect parasite epidemiology and subsequently influence the effectiveness of control. Stochastic models (Renshaw, 1991) can help to evaluate such strategies, by simulating identical scenarios allowing a direct comparison of treatment effectiveness, and to identify potential interactions, thereby aiding in the assessment of the feasibility of novel control strategies. Currently, we are not aware of published simulation models that allow us to account for variation between individual calves within a group and variation in parasite supra population, i.e. parasite populations at all development stages across all hosts.

The aim of this paper was to develop a stochastic simulation model that was capable of accounting for such variation and can be utilized in future studies of parasite control strategies. The stochastic model was based on the deterministic approach previously developed by Berk *et al.* (2016). The deterministic model is able to account for the interactions between gastrointestinal parasites and an individual calf to predict parasite infra-populations, i.e. populations within individual hosts. By introducing variation in growth and resistance traits amongst calves, along with an epidemiological-transmission layer, we aimed to develop a model which considers *O. ostertagi-*calf interactions along with their epidemiological consequences. Following model development, its behaviour was evaluated under simple manipulations such as variations in stocking rate and larval pasture contamination (*PC*). Finally, the model was validated against the prevailing management control strategies, such as reduced stocking rate, the ‘dose and move’ strategy and strategic anthelmintic drenching.

## MATERIALS AND METHODS

A previously developed dynamic, deterministic model (Berk *et al.* 2016) to describe the interactions between gastrointestinal parasites and an individual calf, was extended to a stochastic one for a grazing population/herd of calves. A brief description of the individual calf model is given below, followed by a more detailed description of the additional features incorporated towards the development of a grazing population model. Abbreviations used throughout the paper are defined below and provided in Appendix Table A1.

### Individual calf model

**Fig. 1.**
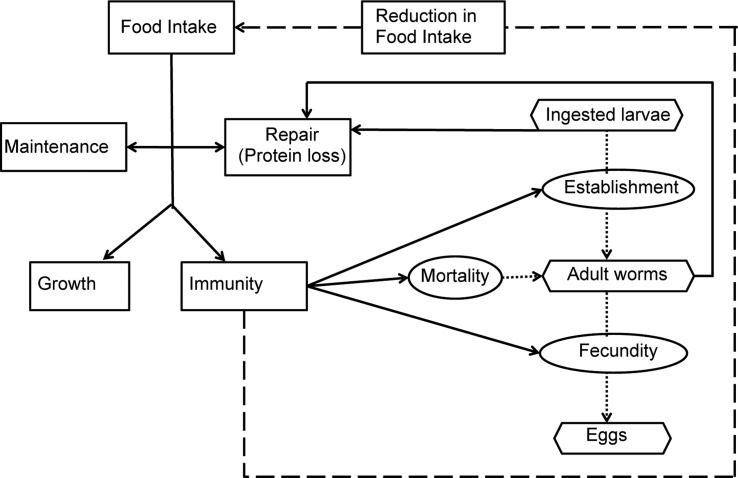
Schematic description of the parasite–host interactions. The rectangular boxes and solid lines indicate the flow of ingested feed resources; the oval boxes indicate the host–parasite interactions and the hexagonal boxes represent the key measurable stages of the parasite life cycle. Host immune response and related pathological and inflammatory responses were assumed to lead to parasite-induced anorexia (broken line).

A schematic diagram representing the model interactions for an individual calf infected by *O. ostertagi* is provided in Fig. 1. Briefly, it was assumed that a healthy calf attempts to ingest sufficient nutrients to meet demands for growth and maintenance (Coop and Kyriazakis, 1999). In the presence of parasitic infection, a parasitized calf experiences an endogenous protein loss (Fox, 1993). Consequently, the calf is assumed to invest in an immune response to reduce the impact of infection (Claerebout and Vercruysse, 2000). However, despite the endogenous protein loss and the increased resource requirement for the development of immunity, a reduction in feed intake occurs as a result of immune components, e.g. cytokines and related pathological and inflammatory responses (Fox *et al.* 1989; Kyriazakis, 2014). This reduction was modelled as a function of the rate of acquisition of immunity (Laurenson *et al.* 2011). Consequently, the calf consumes insufficient food resources to fulfil its requirements. Ingested protein, after the loss due to parasitism, was assumed to be first allocated to maintenance and repair requirements (Coop and Kyriazakis, 1999). Remaining food resources were then allocated between growth and immunity, proportional to their requirements (Kahn *et al.* 2000; Doeschl-Wilson *et al.* 2008; Laurenson *et al.* 2011). Such requirements were defined in accordance to Vagenas *et al.* (2007).

### Herd population model

In contrast to previously published models (Vagenas *et al.* 2007; Doeschl-Wilson *et al.* 2008; Laurenson *et al.* 2012), between-animal variation was only modelled at the phenotypic level, for the sake of simplicity. Phenotypic variation was assumed to occur in animal growth characteristics, maintenance requirements and host immunity to gastrointestinal parasitism.

#### Variation in growth characteristics

A growing calf was described by its empty bodyweight at weaning (*EBW*), protein mass at maturity (*P*_*M*_), a growth rate parameter (B*) and the lipid-to-protein ratio at maturity (*LPR*_*M*_). These parameters were selected to minimize correlation to one another, hence preventing problems that would arise from correlated parameters for stochastic simulations (Symeou *et al.* 2016). Growth was assumed to be driven by protein and lipid retention, with expected growth rates described by adaptations of existing functions (Emmans, 1997; Emmans and Kyriazakis, 1997), such that:
1
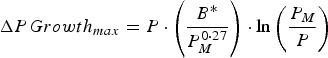

2
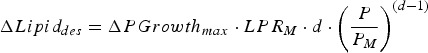

where Δ*PGrowth*_*max*_ is the expected rate of protein retention (kg day^−1^), Δ*Lipid*_*des*_ is the expected rate of lipid retention (kg day^−1^), *P* is the current body protein mass (kg), and 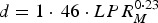.

Thus, differences in initial *EBW* (*EBW*_*i*_), *P*_*M*_, B* and *LPR*_*M*_ can result in between-animal variation in initial body weight, growth rate, mature body composition and mature body weight. As such, these input parameters were assumed to vary phenotypically and are given in Table 1.

**Table 1.** Calf traits for which phenotypic variation between individuals was assumed to occur within the model, with corresponding parameter values for their mean and coefficient of variation (CV)

Category	Parameter (units)	Description	Mean	CV
Growth	*P*_M_ (kg)	Mature protein content	106	0·125
	LRR_M_ (kg)	Mature lipid to protein ratio	1·95	0·15
	B* (day^−1^)	Protein growth rate constant	0·025	0·15
	EBW_i_ (kg)	Initial empty body weight	255	0·15
Maintenance	PR_*maint*_	Coefficient for protein maintenance requirements	0·004	0·15
	ER_*maint*_	Coefficient for lipid maintenance requirements	1·63	0·15
Immunity	*EM*_*max*_ (day^−1^)	Max. combined establishment/mortality	0·82	0·1
	*EM*_*min*_ (day^−1^)	Min. combined establishment/mortality	0·08	0·1
	*μ*_*max*_ (day^−1^)	Max. mortality	0·12	0·2
	*μ*_*min*_ (day^−1^)	Min. mortality	0·01	0·1
	*F*_*max*_ (egg/female/day)	Max. fecundity	39	0·3
	*F*_*min*_ (egg/female/day)	Min. fecundity	6	0·1
	*k*_*EM*_	Rate change parameter for combined establishment/mortality	−2·7 × 10^−8^	0·01
	*k*_*μ*_	Rate change parameter for mortality	4 × 10^6^	0·01
	*k*_*F*_	Rate change parameter for fecundity	−2·9 × 10^−7^	0·01

See the text for sources of parameter values.

#### Variation in maintenance requirements

The body maintenance requirements for protein (*PR*_*maint*_, kg day^−1^) and metabolizable energy (*ER*_*maint*_, MJ day^−1^) were modelled in accordance with Emmans and Kyriazakis (2001):
3
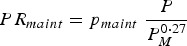

4
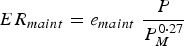

where *p*_*maint*_ is the constant associated with protein maintenance requirements and *e*_*maint*_ is the constant associated with energy maintenance requirements. Phenotypic variation in the parameters *p*_*maint*_ and *e*_*maint*_ was assumed, as it signifies differences in maintenance requirements for protein and energy (Knap and Schrama, 1996; Laurenson *et al.* 2012).

#### Variation in host immunity

The immune response was represented by the host-controlled traits of parasite establishment, mortality (*μ*, proportion of adult worms/day) and fecundity (*F*, eggs/female/day). Establishment was determined by subtracting the effect of mortality from the combined effect of establishment and mortality (*EM*, change in adult worm numbers/day). The functions used to describe these traits were characterized in Berk *et al.* (2016) as:
5
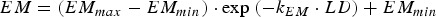

6
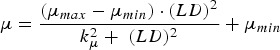

7
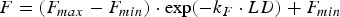

where *LD* is the larvaldays as a measure of parasite exposure; *EM*_*max*_, *μ*_*max*_ and *F*_*max*_ are the maxima of the combined effect of establishment and mortality, mortality and fecundity, respectively; *EM*_*min*_, *μ*_*min*_ and *F*_*min*_ are the minima of the combined effect of establishment and mortality, mortality and fecundity, respectively; *k*_*EM*_, *k*_*μ*_ and *k*_*F*_ are the rate constants of the relationships between larvaldays and the combined effect of establishment and mortality, mortality and fecundity, respectively.

The calves were assumed to be initially naïve to gastrointestinal parasites and gradually acquired immunity as calf exposure to infective larvae increased. The rate of immune acquisition was therefore determined by the length of temporal exposure to infective larvae and the rate parameters *k*_*EM*_, *k*_*μ*_, *k*_*F*_, for each of the host-controlled immunity traits. All parameters describing the maxima, minima and rate of acquisition for each of the host-controlled immunity traits were assumed to exhibit between animal variations.

#### Variation in feed intake

In addition to variation in the specified traits, a degree of random variation was assumed to reflect the influence of external factors controlling variation in day to day feed intake that were not explicitly accounted for by the model. Due to the correlation between growth and feed intake tending towards unity in this model, daily random deviation in feed intake was adjusted to give a more realistic phenotypic correlation between feed intake and growth rate of approximately 0·8 (Cammack *et al.* 2005; Laurenson *et al.* 2012).

#### Parameter values and distributions

The model was parameterized such that the calf and its growth represented a weaned, castrated male (steer) Limousin × Holstein Friesian born in autumn; this common cross currently represents the majority of beef cattle reared in the UK (Todd *et al.* 2011). Autumn born calves are capable of utilizing grass in spring and hence are turned out at 6 months of age and left at pasture until late autumn (Phillips, 2010). Parasitological parameters were based on those gathered from published literature (Berk *et al.* 2016). Each trait selected to be phenotypically variable was assigned a population mean and coefficient of variation (CV) as provided in Table 1 based on several sources. The immune development traits were assumed to follow a log-normal distribution, whereas all other traits were assumed to be normally distributed (Vagenas *et al.* 2007; Laurenson *et al.* 2012). Over recent years calves have been selectively bred to show favourable traits, such as growth rate (Prakash, 2009). However, immune traits are rather more difficult to select for (Frisch, 1981; Prakash, 2009). Log-normal distributions were assigned to the immune rate parameters to allow for higher levels of variation (several-fold increase or decrease) without the negative values that could arise from utilizing a normal distribution for these parameters. For the growth attributes the mean values were taken as presented by Berk *et al.* (2016) and CVs based on estimates for other ruminants (Vagenas *et al.* 2007; Laurenson *et al.* 2012). Similarly, the mean value of immune traits were taken as presented by Berk *et al.* (2016) and, owing to a lack of data to provide confident estimates, CVs were based on values for lambs infected with the closely related parasite *Teladorsagia circumcincta* (Laurenson *et al.* 2012).

All traits, other than those representing the host immune response, were assumed to be uncorrelated (Doeschl-Wilson *et al.* 2008). However, the acquisition of immunity was assumed to be a function of overlapping effector mechanisms (components of the Th2 immune response; Mihi *et al.* 2014). Thus, the rate-determining parameters (*k*_*EM*_, *k*_*μ*_, *k*_*F*_) were assumed to be strongly correlated (coefficient of correlation *r* = +0·5) (Laurenson *et al.* 2012). Establishment was calculated as the combined effect of establishment and mortality minus the effect of mortality alone, as such predictions for establishment and mortality were correlated. In order to counteract this, a negative correlation (*r* = −0·2) was applied to the parameters describing the maximum effect of combined establishment and mortality and the minimum mortality. For correlated traits a Cholesky decomposition of the variance–covariance matrix was used to generate the co-variances between the phenotypic input parameters of the individual animals.

### Epidemiological module

To simulate natural infection of calves in the herd, it was necessary to consider external environmental conditions, including the epidemiology of free-living parasite stages. Many aspects of parasite epidemiology are affected by environmental conditions, in particular temperature and moisture (Stromberg, 1997). Temperature was considered to have the most prominent effect as described below, and moisture was assumed non-limiting under UK conditions. The potential effects of other environmental factors, such as moisture or UV light, were not considered (Stromberg, 1997).

#### Grass quantity and quality

The total grazing pasture available to the calf herd was defined in hectares (*H*, ha). The initial quantity of grass per hectare (*GPH*_0_) was defined as 2500 kg DM ha^−1^ in accordance with English Beef and Lamb Executive (EBLEX) Grazing Planning (2013) and an even grass coverage was assumed. As such, the initial quantity of grass available for grazing (*G*_0_, kg DM) was calculated as:
8
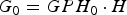


Each day (*t*), the total grass available for grazing (*G*, kg DM) was updated to take into account the grass consumed by the calf population and new grass growth. Thus, *G*_*t*_ was estimated in accordance with Laurenson *et al.* (2012):
9
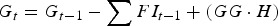

where 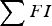 is the total feed intake for all simulated calves, and *GG* is daily grass growth (kg DM ha^−1^) which was estimated for the relevant grazing period using the average grass growth per day for each month reported by EBLEX (2013). *GG* ranged from 30 to 60 kg DM ha^−1^ over the 180 day simulated grazing season.

A reasonably consistent relationship between calendar month and quality of grass has been reported (Trouw Nutrition, 2010; AHDB, 2013). Consequently, the crude protein (*CP*, g kg^−1^ DM) and metabolizable energy (*ME*, MJ kg^−1^ DM) content of grass were time-dependent according to data obtained from fields grazed by cattle in the UK (Woodward *et al.* 1938; Dale *et al.* 2012). As such, over the simulated grazing period of 180 days, *CP* ranged from 165 to 199 g kg^−1^ DM, and *ME* ranged from 11·2 to 12·0 mJ kg^−1^ DM.

#### Pasture contamination

A given number of overwintered infective L_3_ larvae were assumed to be resident on pasture and comprise the initial L_3_ larval contamination (*IL*_0_, L_3_ kg^−1^ DM). As such, the initial total infective L_3_ larval population on pasture (*LP*_0_) was calculated as:
10
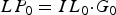


On subsequent days a small number of additional larvae were assumed to become resident on pasture as a result of the maturation and migration of a low level of overwintering eggs, L_1_ and L_2_ (Bairden *et al.* 1995; Urquhart *et al.* 1996). This was modelled as an exponential decay function (Pandey, 1972; Myers and Taylor, 1989), such that the infective L_3_ larvae arising daily from an initial underlying contamination of eggs, L_1_ and L_2_ (*IL*, L_3_ kg^−1^ DM) was estimated on day *t* as:
11
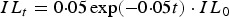


For simplicity, the assumption was that there is a constant relationship between the initial L_3_ contamination and subsequent development of L_3_ from overwinter eggs, L_1_ and L_2_ larvae. However, this consideration was only made prior to the appearance of infective L_3_ larvae arising from eggs deposited by the calf population. The time to earliest appearance of egg-producing adult female worms within the host population, and hence eggs deposited onto pasture, was assumed to be 17 days (Williams *et al.* 1974). The proportion of eggs that develop into infective L_3_ larvae was assumed to be 0·15 (Young and Anderson, 1981). The number of days taken for the eggs to reach the infective L_3_ stage, and the mortality rate of infective L_3_ larvae, were assumed to be temperature dependent (Pandey, 1972; Smith *et al.* 1986).

To model temperature-dependent effects over the simulated grazing season, the mean of the average monthly temperatures observed by the UK Meteorological Office over a 3-year period (2010–2012) were used. A fourth-order interpolating polynomial was fitted to the average monthly temperatures to produce a 6-months temperature curve (Emmanouil *et al.* 2006), such that the maximum temperature (*Temp*, °C) on day *t* was given by:
12
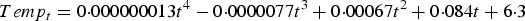


As such, over the simulated grazing period of 180 days, *Temp* ranged from 7·8 to 15·4 °C.

An exponential relationship was fitted between paired data describing temperature and development time (*DT*), i.e. number of days taken to develop from egg to an infective L_3_ larva on pasture (Rose, 1961). As a result, the mean development time of eggs deposited on day *t, DT* (days, rounded to the nearest integer), was assumed to be dependent on *Temp*:
13
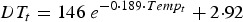


The stochastic nature of development time was represented as a uniform distribution (mean = *DT*_t_ days, range = ±4 days), over whole day increments (Rose, 1961). As such, *DT* ranged from 7 to 40 days over the simulated grazing period. Thus, the number of new infective L_3_ larvae (*newIL*) arising from eggs previously deposited by the calf population was calculated from a convolution of egg deposition and egg maturation time distributions:
14
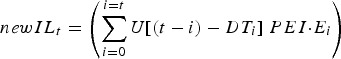

where *U*[*~*] is a uniform probability distribution centred at zero with a range of −4 to +4 days, and *t* is the current day, *i* any previous day (from 0 to current day), *E*_*i*_ the total egg output of the calf population on day *i, DT*_*i*_ the mean development time for eggs deposited on day *i*, and *PEI* the proportion of eggs that develop into infective L_3_ larvae. *U* has a value of ~11% probability of maturing on day *DT* after deposition on pasture, and on the 4 days previous and following day *DT*.

The relationship between *Temp* and the larval mortality rate (*L*_3_*M*, proportion of infective L_3_ larvae dead day^−1^) was defined using data from Young and Anderson (1981) for the temperature ranges observed in the UK. A linear relationship was assumed (Grenfell *et al.* 1986), such that *L*_3_*M* on day *t* was given as:
15
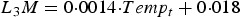


Over the simulated grazing period, *L*_3_*M* ranged from 0·029 to 0·040 (Young and Anderson, 1981).

Consequently, the total infective L_3_ larval population on pasture (*LP*) at the start of day *t* was given as:
16
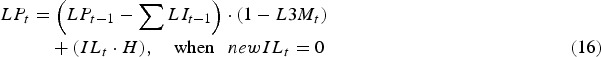

17
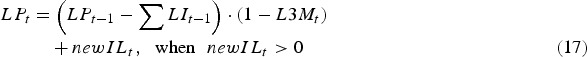

where 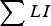 is the total larval intake of the calf population.

#### Larval intake

Calves were assumed to graze randomly across pasture. However, the spatial distribution of the larvae across the pasture was assumed to be aggregated (Boag *et al.* 1989; Grenfell *et al.* 1995; Verschave *et al.* 2015). A negative binomial probability distribution was used with the mean being mean larval contamination of pasture (L_3_ kg^−1^ DM) and the exponent describing the degree of aggregation *k* = 1·41 (Verschave *et al.* 2015). Hence, the larval intake (*LI*, infective L_3_ larvae) of an individual calf was determined by its feed intake (*FI*, kg DM) and by sampling the pasture according to the negative binomial distribution, such that:
18
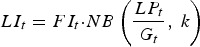

where *LP*_*t*_/*G*_*t*_ (L_3_ larvae day^−1^) is the mean number of L_3_ larvae per ha grazed on day *t*.

### Simulations

The modelled herd comprised 500 calves generated using a stochastic Monte-Carlo simulation, created in MATLAB (2015). For the model inputs defined in Table 1, this population size resulted in a maximum relative s.e. of 1·34% (estimated for *F*_*max*_), which was considered sufficiently large given that further increases in population size showed no further reduction in s.e.

#### Model behaviour

Model behaviour was evaluated by simulating a selection of *IL*_0_ levels and stocking rates. To investigate model behaviour under differing *IL*_0_ levels (0, 100, 200 or 500 *O. ostertagi* L_3_ kg^−1^ DM), the grazing area was set to 100 ha to represent a conventional stocking rate of 5 calves ha^−1^ (EBLEX, 2013). To investigate model behaviour under differing stocking rates, *IL*_0_ was set to 200 *O. ostertagi* L_3_kg^−1^ DM, and the grazing area adjusted for low (3 calves ha^−1^), conventional (5 calves ha^−1^) and high (7 calves ha^−1^) stocking rates, as defined by EBLEX (2013). In all cases, calves were assumed to be parasitologically naïve when turned out in early April for 180 days. Model outputs were calculated on a daily basis and presented as the population mean for: (1) parasite worm burden (*WB*, worms); (2) fecal egg count (*FEC*, eggs g^−1^ feces); (3) feed intake (*FI*, kg DM); (4) relative reduction in calf bodyweight gain (*BWG*, kg) (comparative to a non-parasitized healthy calf); and (5) pasture larval contamination (*PC*, L_3_ larvae kg^−1^ DM).

#### Model validation (control strategies)

To validate model outputs, predictions were compared with observations made in experimental studies investigating the impact of a variety of nematode control strategies (stocking rates, ‘dose and move’ and strategic anthelmintic treatment). Where possible, experimental observations were compared with the population mean for the following model outputs: (1) *FEC* (eggs g^−1^ feces); and (2) *PC* (L_3_ kg^−1^ DM). Where observed percentages of *O. ostertagi* present in relation to other parasites were recorded, direct quantitative comparisons were made. In cases where parasite species differentiation was not made the total numbers of strongyle eggs or pasture larval counts were used to provide a qualitative validation.

Experimental studies from available literature were selected for comparison on criteria stated in Appendix Table A2. A thorough literature review identified the following eight studies that met the specified criteria and were therefore used to validate model predictions for: (1) stocking rate (Nansen *et al.* 1988); (2) strategic dosing (Jacobs *et al.* 1989; Fisher and Jacobs, 1995; Taylor *et al.* 1995; Vercruysse *et al.* 1995; Satrija *et al.* 1996; Sarkũnas *et al.* 1999); and (3) dose and move (Michel and Lancaster, 1970). Initial model input values were taken from each study and included: (1) the initial larval contamination (L_3_ kg^−1^ DM); (2) calf stocking rate; (3) day of turnout; and (4) experimental treatment strategy. For cases where calves received unplanned supplementary feed or emergency anthelmintic treatments part way during the experimental period, measurements taken beyond these points were not included. The actions taken to ensure that the simulations were comparable with experimental observations are below.

#### Growth rates

The model required *P*_*M*_ and B* as inputs. All studies meeting the criteria described above were performed a number of years ago and hence it was necessary to account for changes that may have occurred in these traits as a result of selective breeding. This was done according to the method detailed in Berk *et al.* (2016). It was assumed that calf body composition has remained the same with no direct selection for lean cattle, but rather for heavier mature weights (Emmans and Kyriazakis, 2001; Hays and Preston, 2012).

Following this, the mean of parameter B* (Table 1) was adjusted such that model outputs reflected the growth rates observed for un-infected calves in each study. In the absence of un-infected experimental control groups, calves under a strategic ivermectin treatment were assumed to reflect the growth rate of un-infected calves. For example, in Michel and Lancaster (1970) calves receiving repeated anthelmintic treatments were assumed to reflect growth rates of un-infected calves.

#### Epidemiological components

To account for the variations in turnout date, the date of turnout was used as an input for each experiment. This allowed for seasonal factors such as grass growth, grass quality and temperature-dependent effects to be adjusted accordingly.

#### Mixed Cooperia infections

It was necessary to consider mixed infections of *O. ostertagi* and *Cooperia* due to limitations in the published literature for model validation. Such infections have been observed to cause a greater depression in growth than mono-specific infections (Kloosterman *et al.* 1984; Satrija and Nansen, 1993). It is widely recognized that although in a single *O. ostertagi* infection any protein loss can be reabsorbed in the small intestine, in a mixed infection the presence of *Cooperia* in the small intestine hinders the reabsorption process (Fox, 1993; Holmes, 1993). Thus, parameters describing the protein loss associated with both larval and worm mass were increased by 10% (Kloosterman *et al.* 1984).

#### Control via stocking rate

The constant population size of 500 calves was used for all simulations. As such, the total grazing area (*H*, ha) was adjusted to match the differing stocking rates of each experimental study. In the experimental study of Nansen *et al.* (1988), which investigated two stocking rates, a mid-season rotation was incorporated whereby half of the calves were moved to clean pastures, thus halving the stocking rate on current pasture. To account for this, *H* was doubled at the appropriate time-point. Further, to simulate calves that moved to a clean pasture the same parameters were defined; however, at the time of the mid-season rotation when *H* was increased, the *PC* was also reset to 10 L_3_ kg^−1^ DM as representative of a ‘clean’ pasture.

#### Control via dose and move

During the period for which Michel and Lancaster (1970) conducted their study, ivermectin was not available and thiabendazole was the drug of choice; the efficacy of this drug is likely to have been high at the time of this experiment and hence an efficacy of 0·99 and no residual activity (Prichard *et al.* 1981) were assumed. Following anthelmintic drenching, calves were immediately moved to a ‘cleaner’ pasture by resetting the grass available for grazing (*G*_*t*_) to 2500 kg DM ha^−1^ (EBLEX, 2013) and *PC* to 50 L_3_ kg^−1^ DM (with no resident egg, L_1_ or L_2_ population).

#### Control via strategic anthelmintic treatment

Although there are no universal guidelines for strategic anthelmintic dosing, the recommended timings for administration of ivermectin are 3, 8 and 13 weeks post-turnout in order to minimize worm egg output until mid-July, when most overwintered larvae have died (Cockroft, 2015). Ivermectin, the most widely used anthelmintic, was assumed to have an efficacy of 0·99 against *O. ostertagi* with residual activity for three weeks (NOAH, 2015). Following this period of residual efficacy against *O. ostertagi*, ivermectin efficacy was assumed to decrease by 0·15 per day. This was parameterized such that model predictions for *FEC* and *PC* exhibited similar patterns to those observed in ivermectin treated calves (Vercruysse *et al.* 1988). Ivermectin was assumed to be equally effective against all worm and larval stages residing within the host.

## RESULTS

### Model behaviour

#### Frequency distribution of output traits

**Fig. 2.**
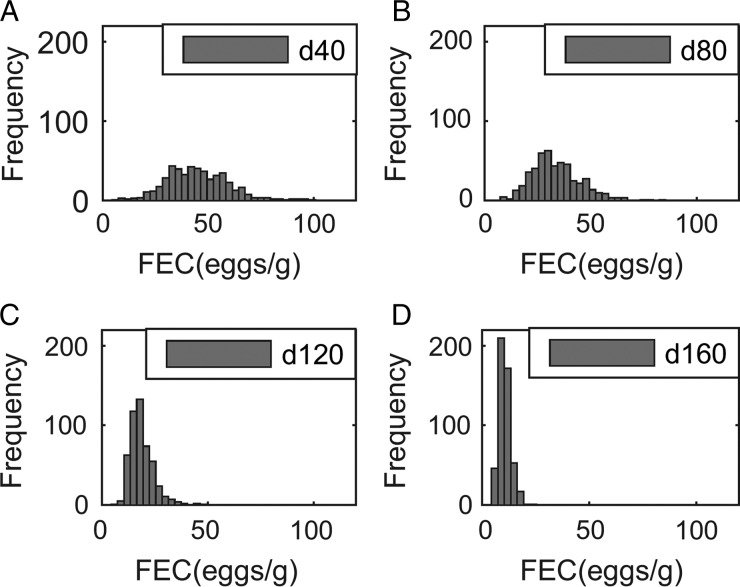
Frequency distribution of fecal egg counts (FEC, eggs g^−1^ feces) of 500 calves grazed at a conventional stocking density of 5 calves ha^−1^ on a pasture initially contaminated with 200 *Ostertagia ostertagi* L_3_ kg^−1^ DM grass, on day: (A) 40, (B) 80, (C) 120 and (D) 160.

Output performance traits were normally distributed at all times. For example, the means (and s.d.) for body weight were 363 (32·7), 429 (41·5), 487 (51·5) and 534 (60·4) kg at 40, 80, 120 and 160 days post-turnout, respectively, for calves grazing clean pasture at a conventional stocking density (5 calves ha^−1^). In contrast, although parasitological inputs were normally or log-normally distributed, the frequency distribution of predicted *WB* and *FEC* became increasingly right-skewed over time, as demonstrated for *FEC* in Fig. 2.

### Increasing initial contamination *(IL_0_)*

#### Parasitological traits

**Fig. 3.**
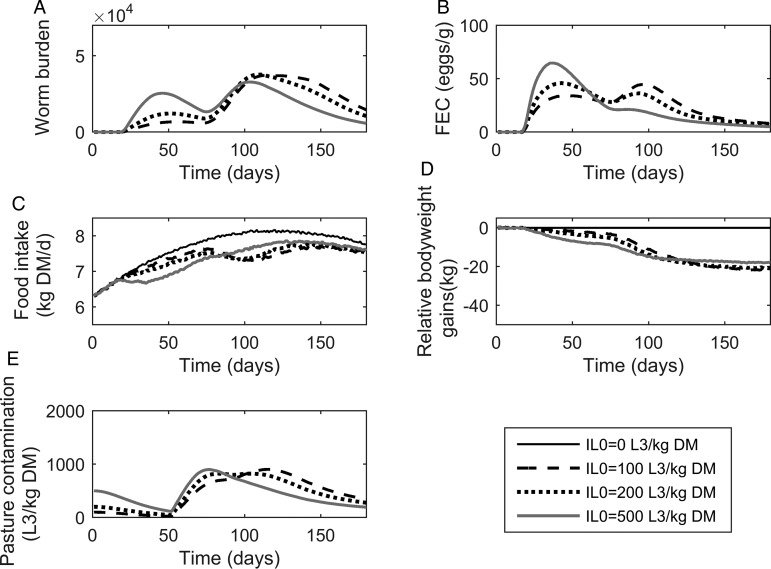
The mean parasitological and performance traits for 500 calves, at a conventional stocking rate of 5 calves ha^−1^, grazing pasture initially contaminated (*IL*_0_) with either 0, 100, 200 or 500 *Ostertagia ostertagi* L_3_ kg^−1^ DM grass. The parasitological traits provided are: (A) mean worm burden and (B) mean fecal egg count (eggs g^−1^ feces) for the population. The performance traits provided are: (C) mean feed intake (kg DM) and (D) mean relative body weight gain (kg) in relation to the un-infected calf population. The epidemiological trait provided is: (E) pasture larval contamination (L_3_ kg^−1^ DM grass).

The population mean of *WB* and *FEC* for *IL*_0_ levels of 100, 200 and 500 L_3_ kg^−1^ DM are given in Fig. 3A and B. Whilst increasing *IL*_0_ caused minor changes in the maximum predicted *WB*, the timing of peak *WB* was predicted to decrease with increasing *IL*_0_. The maximum mean *WB* (and day of peak) for *IL*_0_ levels of 100, 200 and 500 L_3_ kg^−1^ DM were 37 159 (114), 37 772 (109) and 32 831 (103), respectively. For the highest *IL*_0_ of 500 L_3_ kg^−1^ DM an additional small peak in *WB* was observed during the early stages of infection at approximately day 45. Additionally all *IL*_0_ levels showed a marked increase in the gradient of *WB* around day 80. Similar to *WB*, the day of peak *FEC* (eggs g^−1^ feces) decreased with increasing *IL*_0_, and caused minor changes in the maximum predicted *FEC*. The maximum *FEC* (and day of peak) for *IL*_0_ levels of 100, 200 and 500 L_3_ kg^−1^ DM were 47 (95), 48 (43) and 67 (38), respectively. The intermediate *IL*_0_ of 200 L_3_ larvae kg^−1^ DM was predicted to show a similar maximum *FEC* to the lowest *IL*_0_ of 100 L_3_ larvae kg^−1^ DM; however, two peaks of approximately equal magnitude were observed. Ultimately, *FEC* reached similar final levels irrespective of *IL*_0_.

#### Performance traits

The population mean for *FI* and relative reductions in *BWG* are given in Fig. 3C and D. Increasing *IL*_0_ resulted in an increased maximum reduction and earlier achievement of maximum reduction in *FI*, and a faster rate of recovery towards the *FI* of an uninfected calf. Across the duration of the grazing season the average *FI* for control calves on clean pasture was 7·64 kg DM day^−1^: the average relative reductions were 5% for all *IL*_0_ levels. Consistent with the predicted patterns for *FI*, reductions in *BWG* were greater for higher *IL*_0_ in the early stages of infection; however, in the latter stages the magnitude of differences between *IL*_0_ became negliable. The average relative reductions in average daily *BWG* across the season were 0·12, 0·12 and 0·10 kg day^−1^ for *IL*_0_ levels of 100, 200 and 500 L_3_ kg^−1^ DM, respectively.

#### Pasture contamination

Predictions for *PC* (L_3_ kg^−1^ DM) are given in Fig. 3E. Similar patterns were observed for all *IL*_0_ with *PC* decreasing up until day 52 when *PC* began to increase towards a peak. Increasing *IL*_0_ resulted in an earlier peak, however, the maximum predicted *PC* did not relate directly to *IL*_0_. The intermediate *IL*_0_ of 200 L_3_ kg^−1^ DM showed the lowest peak *PC*. The maximum predicted *PC* (and day of maximum) for *IL*_0_ levels of 100, 200 and 500 L_3_ kg^−1^ DM were 903 (116), 825 (82) and 901 (77) L_3_ kg^−1^ DM, respectively. Upon reaching the peak, *PC* then declined to similar levels, irrespective of *IL*_0_.

### Stocking rate

#### Parasitological traits

**Fig. 4.**
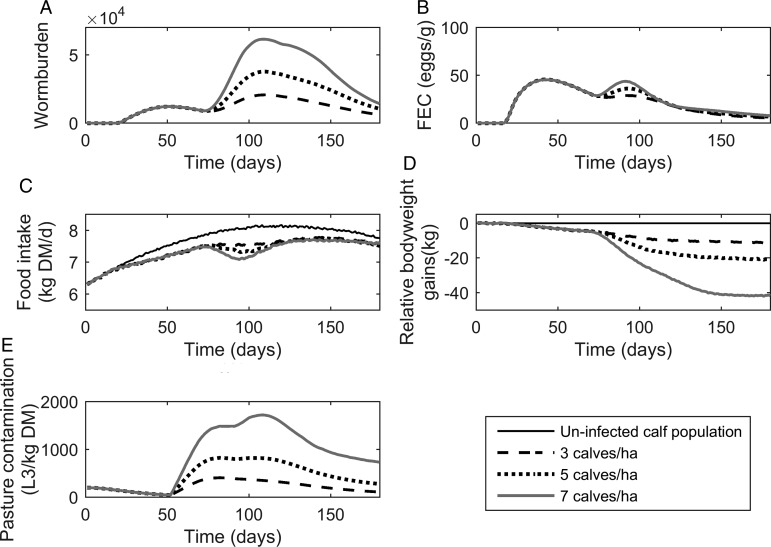
The mean parasitological and performance traits for 500 calves grazing pasture initially contaminated with 200 *Ostertagia ostertagi* L_3_ kg^−1^ DM grass, and kept at stocking rates of either 3, 5 or 7 calves ha^−1^. The parasitological traits provided are: (A) mean worm burden, and (B) mean fecal egg count (eggs g^−1^ feces) for the population. The performance traits provided are: (C) mean feed intake (kg DM) and (D) mean relative body weight gain (kg) in relation to the un-infected calf population. The epidemiological trait provided is: (E) pasture larval contamination (L_3_ kg^−1^ DM grass). The group of untreated calves showed no differences in feed intake and growth due to the assumption of optimal grass availability at the start of the grazing season.

The population mean for *WB* and *FEC* for three stocking rates are given in Fig. 4A and B. Calf stocking rates had no effect on *WB* until day 78, at which point *WB* increased with increasing stocking rates as a reflection of patterns in *PC*. Higher stocking rates resulted in increased maximum *WB*. The maximum *WB* (and day of peak) for low, conventional and high stocking rates were 20 749 (110), 37 772 (109) and 61 508 (109), respectively. Maximum *FEC* was similar for all stocking rates as was the day of FEC peak. The maximum *FEC* (and day of maximum) for low, conventional and high stocking rates were 48 (44), 48 (43) and 48 (38), respectively. A second peak in FEC was observed for conventional and high stocking rates; the second peak (and day of peak) for conventional and high stocking rates were at 38 (94) and 44 (90), respectively.

#### Performance traits

The population mean for *FI* and relative reduction in *BWG* are given in Fig. 4C and D. As with the parasitological outputs, there was no divergence between stocking rates for either of the performance traits until day 78. The maximum reduction in *FI* increased with increasing stocking rates, and *FI* remained compromised in relation to un-infected calves for all stocking rates throughout the simulated grazing period. Across the duration of the grazing season, the average *FI* for control calves on clean pasture was 7·64 kg DM day^−1^, and the average comparative *FI* were reduced by 4, 5 and 5% for low, conventional and high stocking rates, respectively. The relative reduction in *BWG* increased for increasing stocking rates. The average daily *BWG* across the season were reduced in comparison to uninfected calves by 0·07, 0·12 and 0·24 kg day^−1^ for low, conventional and high stocking rates, respectively.

#### Pasture contamination

Predictions for *PC* (L_3_ kg^−1^ DM) are given in Fig. 4E. Initially, similar patterns were observed for all stocking rates with *PC* decreasing until day 52, at which point L_3_ from eggs deposited on pasture eggs first appear and *PC* increased to a peak and then declined. Increasing stocking rates resulted in an increased maximum *PC*. The maximum predicted *PC* (and day of maximum) for low, conventional and high stocking rates were 409 (82), 825 (82) and 1722 (108) L_3_ kg^−1^ DM, respectively. It was therefore observed that *IL*_0_ did not affect performance or infestation significantly.

### Validation

The following sections detail model outputs for the validation simulations.

#### Stocking rates

**Fig. 5.**
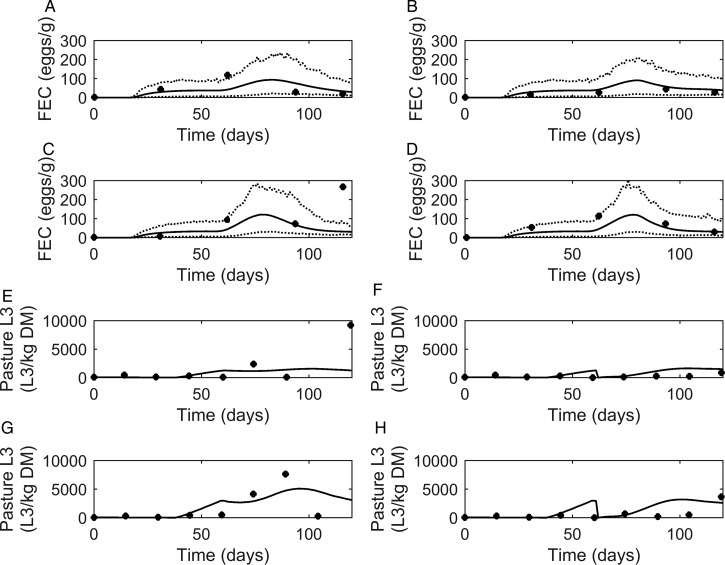
Comparison of experimental observations (●) of Nansen *et al.* (1988) to simulated mean prediction (-) for fecal egg count (*FEC*, eggs g^−1^ feces) (A–D) and pasture contamination (L_3_ kg^−1^ DM grass) (E–H), along with the lower and upper extreme values (^**…**^) for individuals within the simulated population. Calves were kept at a moderate stocking rate (11·7 calves ha^−1^) for the first half of the grazing season, and on day 60, split into two equal groups (5·8 calves ha^−1^) and either: (A) remained on the same pasture or (B) moved to a cleaner pasture (10 L_3_ kg^−1^ DM grass). This was repeated for a high stocking rate (17·5 calves ha^−1^), and on day 60, groups of calves (8·8 calves ha^−1^) either: (C) remained on the same pasture or (D) moved to a cleaner pasture (10 L_3_ kg^−1^ DM grass).

Graphical comparisons of *FEC* between the model and the experiments conducted by Nansen *et al.* (1988) are provided in Fig. 5A–D. In general, model predictions showed similar patterns to the observed data. *FEC* increased steadily to a peak and then began to decline, with the exception of observations made on calves kept at high stocking rates on the same pasture (Fig. 5C), for which a high *FEC* was observed at the final measurement. The majority of data were close to the predicted population mean, and all observations except one were between the estimated lower and upper extreme values of the modelled population.

A graphical comparison for observed and predicted levels of *PC* is provided in Fig. 5E–H. For calves remaining on the same pasture throughout the study (Fig. 5E and G), the model predicted *PC* to increase to a peak and then decline. A slight dip was predicted on day 60 when the stocking rate was halved. For the calves moved to clean pasture on day 60 post-turnout (Fig. 5F and H), the model predicted an increase in *PC* up until day 60 when *PC* was reset to low levels; after which *PC* increased to a peak then slowly declined. For both comparisons of *PC*, a more pronounced effect was seen at the higher stocking rate. Although there was some lack of consistency in the patterns of observed values the model predictions appear to show a reasonable likeness to individual observed points upon graphical comparison, with the exception of the final measurements taken for calves remaining on the same pasture for both stocking rates; the latter appears to be an outlier among the other observations.

#### Dose and move

**Fig. 6.**
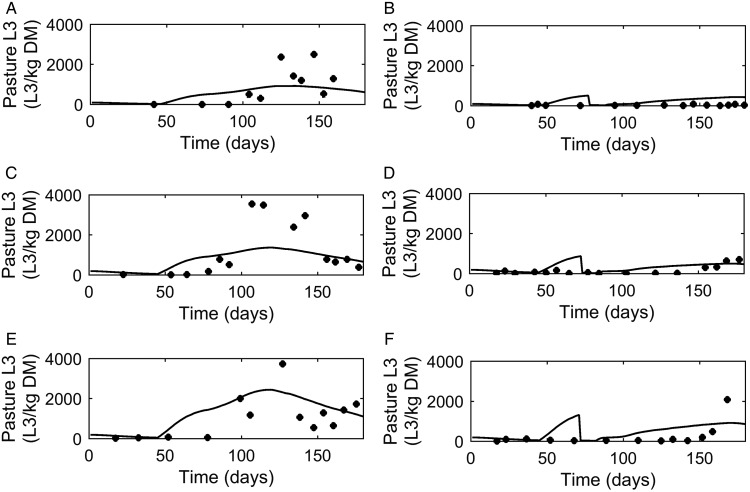
Comparison of experimental observations (●) of Michel and Lancaster (1970) to simulated predictions (-) for pasture contamination (L_3_ kg^−1^ DM grass). For untreated control calves grazed on pasture in: (A) 1965, (C) 1966 and (E) 1967. For calves given thiabendazole on day 70 and moved to ‘clean’ pasture (50 L_3_ kg^−1^ DM grass) in: (B) 1965, (D) 1966 and (F) 1967.

A graphical comparison of *PC* was made for the three dose and move experiments conducted in successive years (Michel and Lancaster, 1970). For calves remaining on the same pasture (Fig. 6A, C and E) similar patterns were seen for observed and predicted outputs with an increase in *PC* up to a peak followed by a decline. The calves moved mid-July (Fig. 6B, D and F) showed a reduced contamination from the move date with only a small increase in *PC* on the new pasture.

#### Strategic dosing

**Fig. 7.**
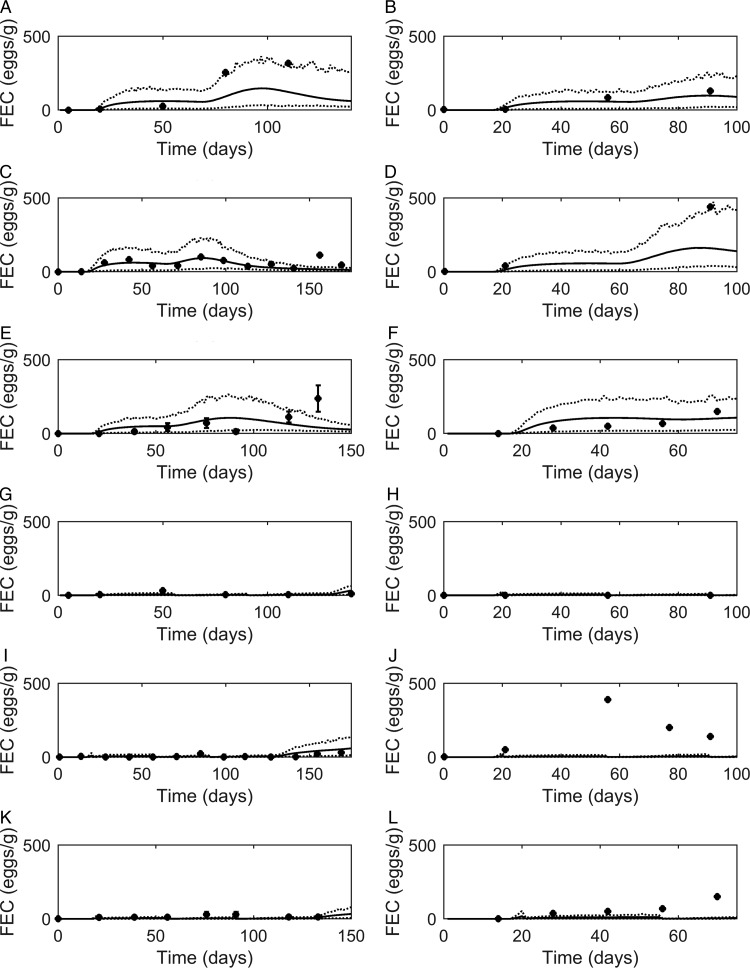
Comparison of experimental observations (●) to simulated mean prediction (-) for fecal egg count (*FEC*, eggs g^−1^ feces), along with the predicted lower and upper extreme values (^**…**^) for individuals within the simulated population. Predictions were made for the group of calves receiving no anthelmintic treatment for experimental data from: (A) Taylor *et al.* (1995), (B) Vercruysse *et al.* (1995), (C) Satrija *et al.* (1996), (D) Fisher and Jacobs (1995), (E) Jacobs *et al.* (1989) and (F) Sarkũnas *et al.* (1999). Comparisons were also made for calves receiving ivermectin on weeks 3, 8 and 13 post-turnout (G–L).

**Fig. 8.**
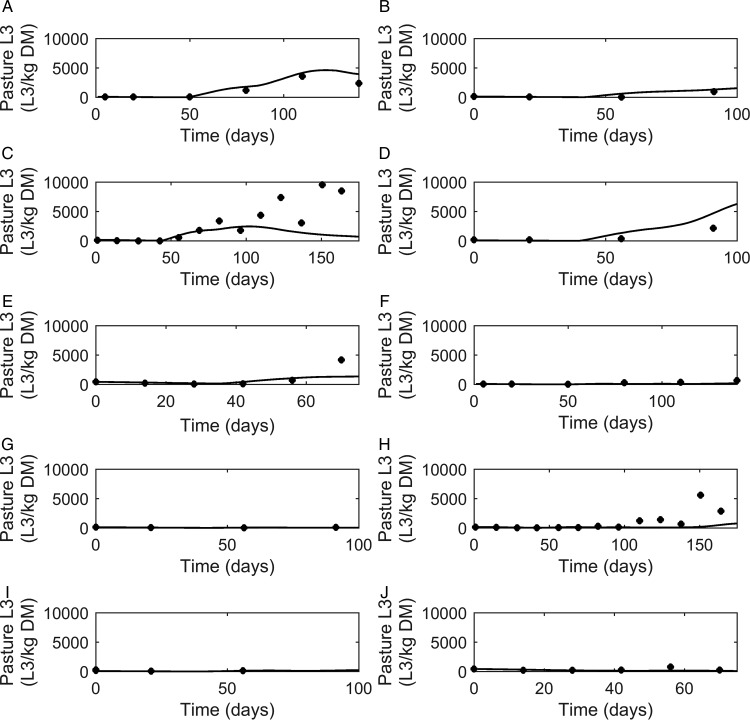
Comparison of experimental observations (●) to simulated mean prediction (-) for pasture contamination (L_3_ kg^−1^ DM grass) in the group of calves receiving no anthelmintic treatment. The experimental data are from: (A) Taylor *et al.* (1995), (B) Vercruysse *et al.* (1995), (C) Satrija *et al.* (1996), (D) Fisher and Jacobs (1995) and (E) Sarkũnas *et al.* (1999). Comparisons were also made for calves receiving ivermectin on weeks 3, 8 and 13 post-turnout (F–J).

Graphical comparisons of *FEC* for each of the six previously identified strategic anthelmintic dosing studies are presented in Fig. 7A–F. Predicted *FEC* in the untreated groups were similar to observed *FEC*. Observed *FEC* increased as time progressed, and in studies conducted for a sufficient time period (>150 days) *FEC* reached a peak and began to decline (Jacobs *et al.* 1989; Taylor *et al.* 1995; Satrija *et al.* 1996) although rebounded later. Model predictions were consistently similar to observations made for the ivermectin treated groups (Fig. 7G–L) which showed low *FEC* across time, with the exception of data from Fisher and Jacobs (1995). For all comparisons, the majority of data were close to the predicted population mean for *FEC*, falling between the estimated lower and upper extreme values for individuals of the modelled population. Additional graphical comparisons were made for *PC* for five of the studies; a graphical comparison for untreated calves is given in Fig. 8A–E, both observed and predicted patterns showed initially an increase in *PC* as time progressed. Congruent with *FEC, PC* also reached a peak and began to decline (Taylor *et al.* 1995). However, this was not supported by Satrija *et al.* (1996), where predictions diverged from observed PC from day 100. For the graphical comparisons of ivermectin treated groups (Fig. 8F–J), all observations and predictions showed a low level of *PC*, with the exception of Satrija *et al.* (1996) where a notable increase in *PC* was observed at the latter stages of the experiment.

## DISCUSSION

A stochastic model was developed to account for the impacts of variation between calves in their ability to deal with *O. ostertagi*, under management conditions that have the potential to affect parasite infra- and supra-populations. Previous comparable studies where calves received the same, or similar, levels of parasite challenge indicated disparities in the immune response exhibited by individuals (Michel, 1969; Michel and Sinclair, 1969). A recent meta-analysis on *O. ostertagi* infections of calves (Verschave *et al.* 2014) found large variations between studies when predicting immune responses. Thus, introducing such variation in simulation models is necessary, as individuals affect parasite epidemiology and can influence the effectiveness of controls. This cannot be captured by models that assume that all individuals within a group are alike and deal with an ‘average’ animal, as is the case for deterministic models (Smith and Guerrero, 1993; Grenfell *et al.* 1995; Fox *et al.* 2013).

Stochastic models enable to address uncertainty and variability in the various factors believed to be important in the behaviour of the system, which in this case comprises the cattle herd, the parasites and their environment. The major issues explored here was variation within the herd and how the distribution of parameter values could affect herd performance and parasitological outputs. The mean characteristics of the system reflect complex interactions of the model parameters, which were defined as probabilistic distributions rather than fixed values. Beyond the mean characteristics, the model also predicted the expected range of outcomes for FEC, such as those shown in Figs 5 and 7. For the purposes of comparability, the simulations presented here were performed on a fixed number of calves (*n* = 500) while stocking density values were set by specifying different values for the grazing area; hence, it was possible to compare directly the predicted averages and extremes. It would be possible to model smaller, more typical, herd sizes, but in this case would be necessary to perform multiple simulations to obtain a proper statistical description of herd characteristics. The emphasis in this work has been on describing variation within the calf population, but the approach can be extended to capture uncertainties in other factors. For example, the historical average temperature profile used here could be replaced by a stochastic representation; multiple simulations over time would then give insights into the range of possible outcomes.

Converting our deterministic model into a stochastic one presented us with two major challenges. The first one was to introduce variation between the individuals of a herd. Values that enable parameterization of the variation between individual calves in growth characteristics exist or at least can be deduced (Ferreira *et al.* 1999; Laurenson *et al.* 2011; Mc Hugh *et al.* 2011). This, however, is not the case for traits that are associated with the ability of hosts to deal with the parasite. For this reason, we resorted to values that have been assumed for sheep (Vagenas *et al.* 2007; Laurenson *et al.* 2011). As there is an increased requirement for characterizing animals for a number of phenotypic and genetic traits (Goddard and Hayes, 2009), the hope is that animal breeders will provide such information for health-related traits, in a manner already done for other animals, such as for resistance to mastitis in dairy cattle (Gernand *et al.* 2012).

The second challenge was to introduce an epidemiological component to the model. Previous attempts to quantify free-living stages of *O. ostertagi* have become increasingly complex (Gettinby and Paton, 1981; Grenfell *et al.* 1987; Smith *et al.* 1987*a*; Chaparro *et al.* 2013; Rose *et al.* 2015). As our focus was on host–parasite interactions we kept this aspect relatively simple. Moisture was assumed to be a non-limiting factor, although in reality rainfall and moisture levels may have a notable effect on aspects of parasite epidemiology (Young and Anderson, 1981). However, the net impact on *PC* levels can be considered to be small due to counteracting mechanisms. For example, heavy rainfall increases larval mortality and accelerates the passage of larvae from pasture downward into the soil reservoir (Al Saqur *et al.* 1982; Gruner *et al.* 1982; Grenfell *et al.* 1986), whilst increased moisture helps the transmission of larvae from fecal pats to herbage by translocation and by splash dispersal (Grønvold and Høgh-Schmidt, 1989; Stromberg, 1997). Only temperature was accounted for in the model, as being the most influential climatological feature on *PC* levels (Stromberg, 1997). Development time (*DT*) for eggs to reach infective L_3_ larvae was dependent on the average daily temperature on the day of excretion alone. A cumulative measure of temperature was not used due to the non-linear relationship between temperature and development, and daily fluctuations in temperature. The sensitivity of the average *DT* to temperature was tested by adding random variation (CV = 0·5) in temperature; however, there was little to no impact on the outputs generated suggesting this to be a fair assumption.

Additionally, demographic stochasticity was incorporated into the model in the form of variation in feed intake and random aggregated distribution of larvae in the pasture. Random variation in calf feed intake impacts on calf growth and the larval intake of an individual, whilst aggregated variation in pasture larvae will influence the larval intake of an individual. Seasonal effects are perceived to impact upon the levels of larval aggregation across pasture; this is an almost ubiquitous feature of parasitic infections due to weather-dependent dispersal patterns of L_3_ larvae from fecal pats. It has previously been observed that significant aggregation was only apparent during particular months, with the level of aggregation correlating to larval numbers (Flota-Bañuelos *et al.* 2013). High *PC* related to low aggregation and low *PC* to high aggregation (Flota-Bañuelos *et al.* 2013; Verschave *et al.* 2015). Although mitigating factors, such as passive dispersal or fecal avoidance behaviours (Hutchings *et al.* 2007) are recognized, an aggregated pasture is still expected (Grenfell *et al.* 1995) and accounted for. The negative binomial is known to provide a good empirical relationship for this overdispersion (Barger, 1987; Boag *et al.* 1989; Fox *et al.* 2013); however, to avoid model complexity the level of aggregation (*k*) was assumed the same for all contamination levels.

Contrary to horizontal aggregation, distribution of larvae along the sward was assumed to be evenly distributed. Due to factors such as distance of larvae from the feces, seasonal variations and vertical migration of larvae, modelling the vertical distribution would be incredibly complex (Pandey, 1974). Often greater proportions of larvae are found lower on herbage; this may have implications for calves kept at high stocking rates where calves graze closer to the base of thee sward. An exaggerated increase in larval uptake can be observed relative to lower stocking rates (Gruner and Sauve, 1982), inducing a more rapid immune acquisition.

An investigation of model behaviour highlighted the importance of interactions between immune acquisition and epidemiology. Parasitological burdens of those individuals that exhibited a slow immune acquisition began to recover earlier than might be expected due to the effect of immunocompetent calves within the herd, which produced fewer eggs, acting to reduce *PC* levels. Increasing levels of initial pasture contamination (*IL*_0_) resulted in earlier peaks in *PC* and parasitological outputs (*WB* and *FEC*) arising from higher parasitic exposure and hence a more rapid immune acquisition. Differences between peak values were marginal due to assumed density-dependent effects on parasite fecundity (Michel *et al.* 1978; Smith *et al.* 1987*b*) and the mid-summer rise in *PC*. The faster immune acquisition by calves exposed to high *IL*_0_ enabled them to counteract the mid-summer rise in L_3_ in comparison to a lower *IL*_0_. This is supported by the hypothesis that turnout date, ultimately defining the degree of immune acquisition prior to the mid-summer rise in *PC*, is perhaps more important than *IL*_0_ (Eysker, 1986; Höglund *et al.* 2013*b*; Taylor *et al.* 2015). The final *PC* and net impact of parasitism on performance was similar for all *IL*_0_ levels; this is in line with a meta-analysis which suggested the relationship between weight gain and *IL*_0_ was insignificant (Shaw *et al.* 1998*b*). However, this is not to say *IL*_0_ levels are not important to consider. When accompanied by different control strategies the *IL*_0_ will likely have an impact on parasitological and performance outcomes.

Changes in stocking rate had comparatively greater parasitological and performance effects than changes in *IL*_0_. The effect is generally inconsequential early in the season due to high grass growth and low *PC*; however as the season progresses grass growth subsides and a mid-summer rise in *PC* occurs (Henriksen *et al.* 1976; Nansen *et al.* 1988). At high stocking rates, the intensity of hosts results in lower grass availability and increased total egg excretion, causing a more dramatic rise in *PC*. Consequently, the peak parasitological outputs increased with increased stocking rate, as observed experimentally (Hansen *et al.* 1989; Thamsborg *et al.* 1998). There was a significant difference predicted in the final net performance of calves kept at each stocking rate. Since it was assumed that pasture availability was non-limiting, this was purely a result of infection. This was in line with experimental work showing significant reductions in mean weight gains for conventional and high stocking rates comparative with a low stocking rate (Hansen *et al.* 1989; Thamsborg *et al.* 1998). Although experimentally it is difficult to ascertain whether these losses resulted from parasitism or a lack of grass availability, Nansen *et al.* (1988) concluded that parasitism was the major cause of poor performance at high stocking rates. The model predicted a reduction in *BWGs* of between 5 and 16%; interestingly meta-analyses conducted on a variety of breeds have shown average reduction in *BWG* of 5·4% (Shaw *et al.* 1997) and 22·7% (Shaw *et al.* 1998*a*) for sub-clinical infections. Although breed may affect observed reductions, it should also be noted these may be slightly larger as a result of concurrent *Cooperia* infections; this is discussed later.

To validate the model, the most common control strategies aiming to reduce the parasitic challenge and burden were identified; these included reduced stocking rate and the Weybridge ‘dose and move’ technique (Michel and Lancaster, 1970). ‘Dose and move’ incorporates a planned move coinciding with an anticipated peak in *PC*, generally mid-July for most of the UK (Smith, 2014). It has previously proved to be a successful control strategy (Michel and Lancaster, 1970; Henriksen *et al.* 1976; Nansen *et al.* 1989; Eysker *et al.* 1998). However, lack of pasture availability has made it increasingly difficult to implement low stocking rates and ‘dose and move’ strategies (Herd, 1988; Shaw *et al.* 1997). The ‘dose and move’ strategy is also believed to accelerate the development of anthelmintic resistance by removing refugia on pasture (van Wyk, 2001). As a result, strategic anthelmintic dosing at specific time points has become critical to maintaining calf health. The objective is to prevent the build-up of *PC* by limiting fecal egg output during the early part of the grazing season (Vercruysse and Claerebout, 1997). This is achieved by strategic treatment with anthelmintics, which has been observed to be effective against parasitic gastroenteritis for a full season, under conditions where the parasitic challenge is large enough to induce severe parasitic gastroenteritis in controls (Hollanders *et al.* 1992; Vercruysse *et al.* 1995).

Previous quantitative evaluation of the deterministic model on which the current stochastic one was based, revealed the former model as reasonably proficient at estimating mean parasitological traits. This places a degree of confidence on the current model, provided that its sources of stochastic variation have been estimated accurately. Based on comparing observed and predicted *FEC* for the current, stochastic model in order to estimate parameter values for calf variation and parasite epidemiology, the model appeared to be proficient at estimating observed outputs under the specified scenarios. In cases where discrepancies between predicted and observed *FEC* were observed, contributory factors were identified. Some studies did not distinguish between parasite genera, stating only that *O. ostertagi* were the most prevalent species, whilst in others calves were treated with anthelmintics on clinical grounds following the final measurements used for validation suggesting disease may have been border-line clinical at the time of measurements.

Additional comparisons were made between observed and predicted values for average *PC*; in most cases the predictions provided a good fit, however a few discrepancies were apparent. As previously mentioned, the aggregated nature of *PC* is likely to influence the sampling of *PC*; if sufficient repeated measures are not taken then an under or overestimation of the PC level may occur (Verschave *et al.* 2015). Upon sampling *PC* some experimenters have opted to consciously avoid fecal pats, where the highest concentrations of larvae exist: this may have resulted in an under estimation of observed *PC* (Henriksen *et al.* 1976; Nansen *et al.* 1988). Poor grass growth causes a higher concentration of larvae on pasture (Vercruysse *et al.* 1995) and, as for *FEC,* the lack of distinction between parasite genera may also result in discrepancies between observed and predicted *PC*. A clear example comes from Satrija *et al.* (1996) whereby *PC* switches from predominantly *O. ostertagi* to predominantly *Cooperia* in August; from this point onwards the model does not predict *PC* well.

Should these factors not account for the differences between observed and predicted *PC* it may be a result of a model oversimplification. These may result in inaccurate predictions made on *PC* which in turn would affect the larval intake due to the self-proliferating nature of the relationships defined in the model. If this is the case, explanations for why FEC still provide a good fit must be considered, implying that the within-host relationships may over or under compensate for these differences.

Monospecific and concurrent artificial infections of *O. ostertagi* and *Cooperia* suggested an absence of inter-species interactions (Kloosterman *et al.* 1984; Satrija and Nansen, 1993; Hilderson *et al.* 1995). Concurrent infections did, however, show greater than additive FEC in comparison with the two monospecific infections (Kloosterman *et al.* 1984; Satrija and Nansen, 1993; Hilderson *et al.* 1995), thought to be a consequence of enhanced pathological effects (Parkins *et al.* 1990). This has been suggested to reflect the fact that *Cooperia* increases the rate of protein loss leading to a reduced growth rate and growth requirements. Slower growth will be accompanied by lower feed intake, which will have a concentration effect on FEC due to lower output of feces (Parkins *et al.* 1990). This is supported by reduced pepsinogen levels, reflecting abomasal damage (Parkins *et al.* 1990), and almost doubled plasma losses for concurrent infections comparative to monospecific *O. ostertagi* infections (Kloosterman *et al.* 1984; Parkins and Holmes, 1989). To account for a mixed infection the most comprehensive method would be to create a model component for predicting the effects of *Cooperia* on the host, and determine species interactions. Although some data exists on artificial *Cooperia* infections as has been summarized by Verschave *et al*. (2016), there is very limited data on artificial mixed infections and hence it would be difficult to decipher species interactions for a full range of infection levels.

The development of a stochastic model to account for host–parasite interactions opens up a number of opportunities for future developments. Firstly, it enables the effectiveness of different control strategies to be assessed, including targeted selective treatments where specific individuals of a population are treated, as opposed to the whole population (Höglund *et al.* 2013*a*; O'Shaughnessy *et al.* 2015). This method has been advocated as a potential way to reduce parasite resistance to anthelmintics, but hard, non-confounded data to support this does not exist (Höglund, 2010). Introduction of potential parasite resistance mechanisms would allow for such refugia-based strategies to be assessed for effectivity and sustainability over short- and long-term periods; this would provide a useful tool considering the challenges of experimentally investigating long-term effects. Further to this, the addition of second grazing season (SGS) calves would allow exploration of the impact of different control strategies on the immune acquisition of SGS calves and effects of hypobiosis. The model is also flexible enough to allow the investigation into the consequences of breeding for parasite resistance through the addition of a genetic component. Although breeding of resistant cattle stock would prove challenging (Kloosterman *et al.* 1978) there is large potential for genetic progress, more so than sheep (Kloosterman *et al.* 1992).

## APPENDIX

**Table A1.** A table of common abbreviations used throughout the text

Abbreviation	Definition
BW	Bodyweight
BWG	Bodyweight gain
DM	Dry matter
EBW	Empty bodyweight
FEC	Fecal egg count
FI	Feed intake
H	Hectares
*IL*_0_	Initial pasture contamination
LI	Larval intake
PC	Pasture contamination
WB	Worm burden

**Table A2.** A list of the required criteria that were achieved by experimental studies in order for them to be appropriate for use in validating the model

Criteria	
1	The only available feed was grass
2	The experiment was conducted on calves grazing in spring months and maintained in a temperate environment
3	All calves were infected during the growing phase
4	No calves had exposure to parasites prior to the experiment (i.e. first grazing season calves)
5	Infections were either single *O. ostertagi* or mixed with *Cooperia* spp. (due to the lack of single species *O. ostertagi* infections in literature it was necessary to consider mixed infections; the consequences of *Cooperia* infections were accounted for as described in the main text)
6	Any dosing with ivermectin was administered at the recommended dose of 200 *µ*g kg^−1^ by subcutaneous injection
7	Any dosing with thiabendazole was administered orally at the recommended dose of 200 mg kg^−1^
